# P-1293. Healthcare Costs Associated with Lyme Disease in a US Insured Population

**DOI:** 10.1093/ofid/ofae631.1474

**Published:** 2025-01-29

**Authors:** Rebecca M Fee, Holly Yu, L Hannah Gould, Amanda R Mercadante, Brian T Campfield, John Halperin, Mary G Johnson, Benjamin Chastek

**Affiliations:** Optum, Eden Prairie, Minnesota; Pfizer Inc., Collegeville, Pennsylvania; Pfizer Vaccines, New York, New York; Pfizer, Inc., New York, New York; Division of Pediatric Infectious Diseases, Department of Pediatrics, University of Pittsburgh School of Medicine, Pittsburgh, PA; UPMC Children’s Hospital of Pittsburgh, Pittsburgh, PA; Richard K. Mellon Institute for Pediatric Research, Pittsburgh, PA; Overlook Medical Center, Summit, New Jersey; Optum, Eden Prairie, Minnesota; Optum, Eden Prairie, Minnesota

## Abstract

**Background:**

An estimated 476,000 people are diagnosed and treated for Lyme disease (LD) each year in the United States (US). LD manifests as localized (e.g., single erythema migrans (EM)) or disseminated disease (e.g., multiple EM, arthritis, cardiac, and neurologic conditions). Healthcare costs for LD patients by clinical manifestation are understudied. This work aimed to assess costs of LD by manifestation in an insured US population.

**Figure:**
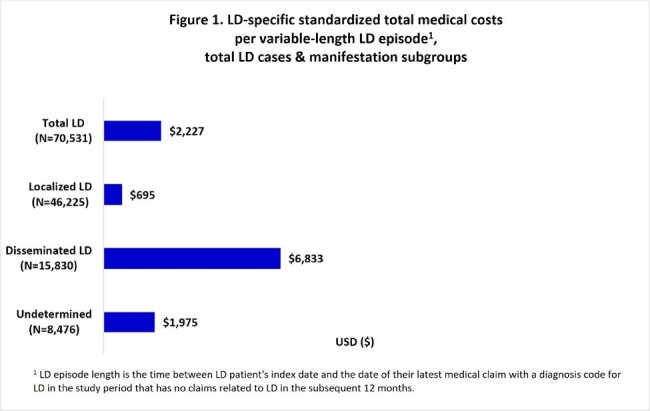

**Methods:**

This retrospective study identified LD patients in the Optum Market Clarity Integrated Claims and Clinical dataset from January 2016 through December 2022 based on LD diagnosis codes (A69.xx). Outpatient LD patients had claims for relevant antibiotics within 30 days of diagnosis. Inpatient LD patients had LD as a primary or secondary diagnosis with a LD-associated condition as primary. Cases were classified as disseminated, localized, or undetermined based on diagnosis codes for LD and LD-associated conditions, inpatient vs. outpatient services, and antibiotic treatment. Healthcare costs were assessed for 1) LD-specific costs during individual LD episodes, 2) all-cause 6-month follow-up costs vs. baseline costs for LD patients, 3) all-cause 6-month follow-up costs for LD vs. controls (matched 3:1 to cases by hard matching and propensity score matching on demographic and clinical characteristics). Costs were standardized across payer types and CPI-adjusted to 2022.

**Figure:**
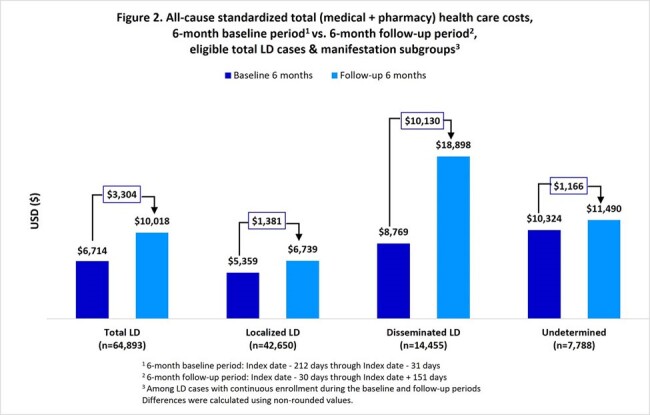

**Results:**

During 2016-2022, 70,531 LD cases (mean age 45 years, 51% female) were identified. Mean (standard deviation) LD-specific total medical costs were $2,227 ($15,790) per episode, with $695 ($7,348) and $6,833 ($27,983) for localized and disseminated cases, respectively (Figure 1). Compared with a 6-month baseline, mean all-cause total costs during 6-month follow-up were $3,304 greater for LD diagnosed patients overall, $1,381 greater for localized, and $10,130 greater for disseminated (Figure 2). Compared to controls, mean total all-cause 6-month follow-up costs were greater by $4,098 for LD overall, $819 for localized, and $12,978 for disseminated (Figure 3).

**Figure:**
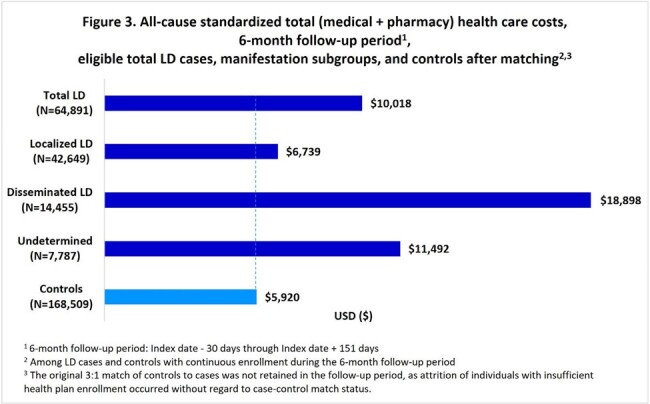

**Conclusion:**

Patients diagnosed with LD incurred substantial costs, driven by disseminated disease. This poses a large economic burden to the healthcare system. Effective preventive measures are needed.

**Disclosures:**

**Rebecca M. Fee, MPH**, Optum: Employee **Holly Yu, MSPH**, Pfizer: Employee|Pfizer: Stocks/Bonds (Public Company) **L. Hannah Gould, PhD, MS, MBA**, Pfizer: Employee|Pfizer: Stocks/Bonds (Public Company) **Amanda R. Mercadante, PharmD**, Pfizer, Inc.: Stocks/Bonds (Public Company) **Brian T. Campfield, MD**, Pfizer: Advisor/Consultant **John Halperin, MD**, Abbott: Stocks/Bonds (Public Company)|Abbvie: Stocks/Bonds (Public Company)|Johnson & Johnson: Stocks/Bonds (Public Company)|Merck: Stocks/Bonds (Public Company)|Pfizer: Advisor/Consultant **Mary G. Johnson, PharmD, MSPH**, UHG/Optum: Employee|UHG/Optum: Stocks/Bonds (Public Company) **Benjamin Chastek, MS**, Optum: Employee|Optum: Stocks/Bonds (Public Company)

